# Corticosteroid therapy versus physiotherapy on pain, mobility and function in shoulder impingement: A short note

**DOI:** 10.4102/sajp.v78i1.1794

**Published:** 2022-12-12

**Authors:** Natalie Benjamin-Damons, Naeema A.R. Hussein El Kout, Rogier van Bever Donker, Tamsen Edwards, Gillian Ferguson

**Affiliations:** 1 Department of Physiotherapy, Faculty of Health Sciences, University of the Witwatersrand, Johannesburg, South Africa; 2 Professional Development Portfolio, The South African Society of Physiotherapy, Johannesburg, South Africa; 3 Department of Physiotherapy, Faculty of Health Sciences, University of KwaZulu-Natal, Durban, South Africa; 4 Department of Physiotherapy, Faculty of Health Sciences, University of Cape Town, Johannesburg, South Africa

**Keywords:** shoulder pain, corticosteroid injections, physiotherapy, evidence statement, subacromial impingement syndrome

## Abstract

**Background:**

The global estimate of shoulder pain is 67% and is often associated with subacromial impingement syndrome. Interventions include corticosteroid injection (CSI) therapy and physiotherapy. Further information is needed to compare the effect of these interventions on pain, joint range of motion (ROM) and shoulder function.

**Objectives:**

To summarise the best evidence comparing the effect of CSI versus physiotherapy on pain, shoulder ROM and shoulder function in patients with subacromial impingement syndrome.

**Method:**

This evidence statement is based on a systematic review and meta-analysis of three randomised controlled trials (RCTs), namely, Rhon et al. (2014) (*n* = 136), Hay et al. (2003) (*n* = 207) and Van der Windt et al. (1998) (*n* = 109), with a total of 452 participants. A total of 14 studies were reviewed and only 3 studies met the inclusion criteria.

**Results:**

An improvement in shoulder function was found in favour of CSI at 6- to 7-week follow-up (*p* < 0.0001), but no evidence was found for the superiority of CSI compared to physiotherapy for pain and ROM over 4–12 weeks. In 24 and 48 weeks, no evidence was found for the superiority of CSI compared to physiotherapy for shoulder function, pain or ROM.

**Conclusion:**

No evidence was found for the superiority of CSI compared to physiotherapy for pain and ROM in the short term besides an improvement in shoulder function in favour of CSI at 6–7 weeks. There was a weak recommendation with moderate quality of evidence based on three RCTs (2B).

**Clinical implications:**

This evidence statement may inform clinical practice when determining which intervention is best suited to manage patients with shoulder pain.

## Background

Shoulder pain because of inflammatory conditions is among the most common complaints globally, with a high burden of disease associated with it (Rahavard, Knezevic & Candido 2020). The prevalence of shoulder pain is estimated to be 67% in the general population (Luime et al. 2004). Hodgetts and Walker (2021) found that only half of shoulder pain cases completely recover after six months.

Shoulder pain often results in decreased functional ability because of a reduction in joint range of motion (ROM; Consigliere et al. 2018). Subacromial impingement syndrome is often found as the most common cause of pain (Cuff & Littlewood 2018). Impingement syndrome refers to the narrowing of the subacromial space, leading to impingement of the tendons of the rotator cuff muscles (Dhillon 2019). Management strategies include inflammatory-reducing pharmacological interventions and physiotherapy techniques such as soft tissue mobilisation, exercise and electrotherapy (Creech & Silver 2021).

## Question definition

*Population*: Male and female adults (18–65 years) with symptoms of moderate or severe unilateral shoulder pain primarily because of inflammatory causes resulting in impingement syndrome, which may require corticosteroid injections (CSI), worsened by movement and having a noncapsular pattern of restriction.

*Intervention:* Subacromial CSI of the affected shoulder.

*Comparison*: Physiotherapy management that had to include a combination of passive and active joint and soft tissue mobilisation techniques.

Outcome measures:

- *Pain*: Measured with the Visual Analogue Scale (Hay et al. 2003; Van der Windt et al. 1998) and the Numeric Pain Rating Scale (Rhon, Boyles & Cleland 2014).
- *Range of motion* (ROM): The range of glenohumeral movements was assessed.
- *Shoulder function*: Assessed with the global rating of change scale (Rhon et al. 2014) and the shoulder disability questionnaire (Hay et al. 2003; Van der Windt et al. 1998).

## Evidence summary

This evidence-based statement was developed from the published article ‘Effect of CSI versus physiotherapy on pain, shoulder ROM and shoulder function in patient with subacromial impingement syndrome: A systematic review and meta-analysis’ (Burger et al. 2016). This evidence statement is based on a systematic review and meta-analysis of three randomised controlled trials (RCTs), namely Rhon et al. (2014) (*n* = 136), Hay et al. (2003) (*n* = 207) and Van der Windt et al. (1998) (*n* = 109), with a total of 452 participants. The composition and site of the CSI differed among studies, as did the physiotherapy management. The three RCTs included exercise and manual therapy. In addition to this, ultrasound was utilised (Hay et al. 2003), and other electrotherapy modalities were also used, which were not specified but excluded ultrasound (Van der Windt et al. 1998). Physiotherapy sessions were conducted over a period of between 6 and 12 weeks, with 20- to 30-min sessions weekly. The physiotherapy treatment interventions in the included RCTs involved, yet were not limited to, the following techniques: contract-relax techniques, manual stretching, exercises aimed at shoulder girdle and spinal strengthening, patient education and electrotherapeutic modalities. The frequency ranged from eight 20-min sessions for 6 weeks, compared to twelve 30-min sessions for 12 weeks, compared to six sessions twice weekly for 6 weeks.

This review reported a significant improvement of shoulder function in favour of CSI at a 6- to 7-week follow-up (*p* < 0.0001), but no evidence was found for the superiority of CSI compared to physiotherapy for pain and ROM over the short term (1–3 months). In the medium term (6 months) and long term (12 months), no evidence was found for the superiority of CSI compared to physiotherapy for either shoulder function, pain or ROM.

## Quality of evidence

The most current evidence was searched for in the literature using the same keywords and database as the original article. In total, 14 full-text studies were reviewed, and only three studies met the inclusion criteria. The authors do acknowledge the limitations with regard to the number of studies included. All three RCTs were of high quality with minimal loss to follow-up, narrow confidence intervals and high PEDro scores. The three RCTs that were included in the meta-analysis of the systematic review were classified as evidence Level II according to the National Health and Medical Research Council (NHMRC 2009) and were all of high methodological quality, scoring an average of 7.3/10 on the Physiotherapy Evidence Database (PEDro) scale. However, the overall evidence from the systematic review is graded moderate in quality, as there is indirectness of treatment or intervention.

The heterogeneity of the study was assessed utilising the *I*^2^ statistic, which was 84%, indicating substantial heterogeneity as a score between 50% and 90% is considered high (Imrey 2020). Because of heterogeneity among the results reported by the three studies for pain and ROM, statistical pooling was determined to be inappropriate, and thus, results were summarised in narrative form.

## Best estimates

The findings suggest that physiotherapy and CSI both showed improvement in pain, shoulder ROM and shoulder function in the short term (1–3 months), medium term (6 months) and long term (12 months) in patients with primary symptoms of moderate or severe unilateral shoulder pain. No evidence was found for the superiority of CSI compared to physiotherapy for pain and ROM in the short term besides a significant improvement in shoulder function in favour of CSI at short-term follow-up. The medium-term and long-term outcomes for pain, ROM and shoulder function show no significant difference between the use of CSI and physiotherapy.

## Judgement of benefits versus risks, burden and cost

Information available suggests that either physiotherapy management or CSI would be beneficial for the treatment of subacromial impingement syndrome resulting in pain and decreased shoulder ROM and shoulder function, and therefore the patient’s preference should be considered when deciding on the management strategy, such as pharmacological intervention or physiotherapy. In addition, physiotherapy is recommended as the low-risk option because of the paucity of evidence for the long-term effects of CSI on subacromial impingement syndrome. It is also important to note the adverse effects versus benefits of CSI, especially with long-term usage. This includes considering benefits such as short-term pain reduction and possible adverse effects such as a compromised immune system and articular cartilage toxicity (Stone, Malanga & Capella 2021). In addition to this, it should be noted that the improvement at 6–7 weeks may be preferable for some patients even though long-term outcomes seem to be similar.

## Grade of recommendation

There was a weak recommendation with moderate quality of evidence based on three RCTs (2B). This may change because of the paucity of available studies.
